# Changes in Media Reporting Quality and Suicides Following National Media Engagement on Responsible Reporting of Suicide in Canada: Changements de la Qualité des reportages dans les médias sur les suicides suite à l’engagement des médias nationaux à la déclaration responsable du suicide au Canada

**DOI:** 10.1177/07067437231223334

**Published:** 2024-01-04

**Authors:** Mark Sinyor, Daniella Ekstein, Nivetha Prabaharan, Lisa Fiksenbaum, Caroline Vandermeer, Ayal Schaffer, Jane Pirkis, Marnin J. Heisel, Benjamin I. Goldstein, Donald A. Redelmeier, Paul Taylor, Thomas Niederkrotenthaler

**Affiliations:** 1 Department of Psychiatry, 71545 Sunnybrook Health Sciences Centre, Toronto, Canada; 2 Department of Psychiatry, 7938 University of Toronto, Toronto, Canada; 3 Factor-Inwentash Faculty of Social Work, 7938 University of Toronto, Toronto, Canada; 4 Department of Psychology, 7991 York University, Toronto, Canada; 5 5116 Viterbi School of Engineering, University of Southern California, Los Angeles, USA; 6 Centre for Mental Health, Melbourne School of Population and Global Health, University of Melbourne, Melbourne, VIC, Australia; 7 Department of Psychiatry, The University of Western Ontario, London, Canada; 8 Centre for Youth Bipolar Disorder, 7978 Center for Addiction and Mental Health, Toronto, Canada; 9 Institute for Clinical Evaluative Sciences, Toronto, Canada; 10 Department of Medicine, 7938 University of Toronto, Toronto, Canada; 11 Division of General Internal Medicine, 71545 Sunnybrook Health Sciences Centre, Toronto, Canada; 12 71545 Sunnybrook Health Sciences Centre, Toronto, Canada; 13 Unit Suicide Research and Mental Health Promotion, Department of Social and Preventive Medicine, Centre for Public Health, Medical University of Vienna, Vienna, Austria; 14 Wiener Werkstaette for Suicide Research, Vienna, Austria

**Keywords:** suicide, media, media reporting, Werther effect, Papageno effect, media guidelines, narratives, suicide, médias, reportages des médias, effet Werther, effet Papageno, lignes directrices médiatiques, récits

## Abstract

**Objective:**

Responsible media reporting is an accepted strategy for preventing suicide. In 2015, suicide prevention experts launched a media engagement initiative aimed at improving suicide-related reporting in Canada; its impact on media reporting quality and suicide deaths is unknown.

**Method:**

This pre–post observational study examined changes in reporting characteristics in a random sample of suicide-related articles from major publications in the Greater Toronto Area (GTA) media market. Articles (*n* = 900) included 450 from the 6-year periods prior to and after the initiative began. We also examined changes in suicide counts in the GTA between these epochs. We used chi-square tests to analyse changes in reporting characteristics and time-series analyses to identify changes in suicide counts. Secondary outcomes focused on guidelines developed by media professionals in Canada and how they may have influenced media reporting quality as well as on the overarching narrative of media articles during the most recent years of available data.

**Results:**

Across-the-board improvement was observed in suicide-related reporting with substantial reductions in many elements of putatively harmful content and substantial increases in all aspects of putatively protective content. However, overarching article narratives remained potentially harmful with 55.2% of articles telling the story of someone's death and 20.8% presenting an other negative message. Only 3.6% of articles told a story of survival. After controlling for potential confounders, a nonsignificant numeric decrease in suicide counts was identified after initiative implementation (ω = −5.41, *SE*  =  3.43, *t*  =  1.58, *p*  =  0.12).

**Conclusions:**

We found evidence that a strategy to engage media in Canada changed the content of reporting, but there was only a nonsignificant trend towards fewer suicides. A more fundamental change in media narratives to focus on survival rather than death appears warranted.

## Abrégé

**Objectif:**

Le reportage médiatique responsable est une stratégie convenue pour prévenir le suicide. En 2015, les experts de la prévention du suicide ont lancé une initiative d’engagement des médias visant à améliorer les reportages liés au suicide au Canada; dont l’effet sur la qualité des reportages médiatiques et sur les décès par suicide n’est pas connu.

**Méthode:**

La présente étude observationnelle pré-post a examiné les changements des caractéristiques des reportages dans un échantillon aléatoire d’articles liés au suicide des principales publications du marché des médias de la Région du Grand Toronto (RGT). Parmi les articles (n  =  900), il y en avait 450 pendant la période étalée 6 ans avant et après le début de l’initiative. Nous avons aussi examiné les changements des nombres de suicides dans la RGT entre ces époques. Nous avons utilisé des tests du chi carré pour analyser les changements de caractéristiques des reportages, et des analyses de séries chronologiques ont servi à identifier les changements des nombres de suicides. Les résultats secondaires portaient sur les lignes directrices élaborées par les professionnels des médias du Canada et sur la manière dont elles ont pu influencer la qualité des reportages médiatiques ainsi que sur la narration globale des articles médiatiques durant les années les plus récentes où les données étaient disponibles.

**Résultats:**

Une amélioration générale a été observée dans les reportages sur le suicide ainsi que des réductions substantielles dans bien des éléments du contenu potentiellement nocif et des augmentations substantielles dans tous les aspects du contenu possiblement protecteur. Toutefois, en globalité, les récits des articles demeuraient possiblement nocifs avec 55,2% des articles racontant l’histoire du décès de quelqu’un et 20,8% présentant un autre message négatif. Seulement 3,6% des articles relataient une histoire de survie. Après un contrôle pour des confondants potentiels, une diminution numérique non significative des nombres de suicides a été identifiée après la mise en œuvre de l’initiative (*ω*  =  −5,41, *SE*  =  3,43, *t*  =  1,58, *p*  =  0,12).

**Conclusions:**

Nous avons constaté la preuve qu’une stratégie d’engager les médias au Canada changeait le contenu des reportages, mais qu’il n’y avait qu’une tendance non significative à la réduction du nombre de suicides. Un changement des récits médiatiques qui seraient axés sur la survie plutôt que sur le décès semble justifié.

## Introduction

Media reports can impact suicide rates through social learning (i.e., imitation effects).^1–14^ The phenomena whereby deaths by suicide increase following well-publicized stories of suicide and decrease following well-publicized stories of survival are termed the “Werther” and “Papageno” effects, respectively.^7,8,15–17^ Our group previously confirmed that Werther and Papageno narratives disseminated in Toronto were associated with changes in suicide counts in the weeks immediately following publication, in the expected directions.^18^

**Figure 1.**
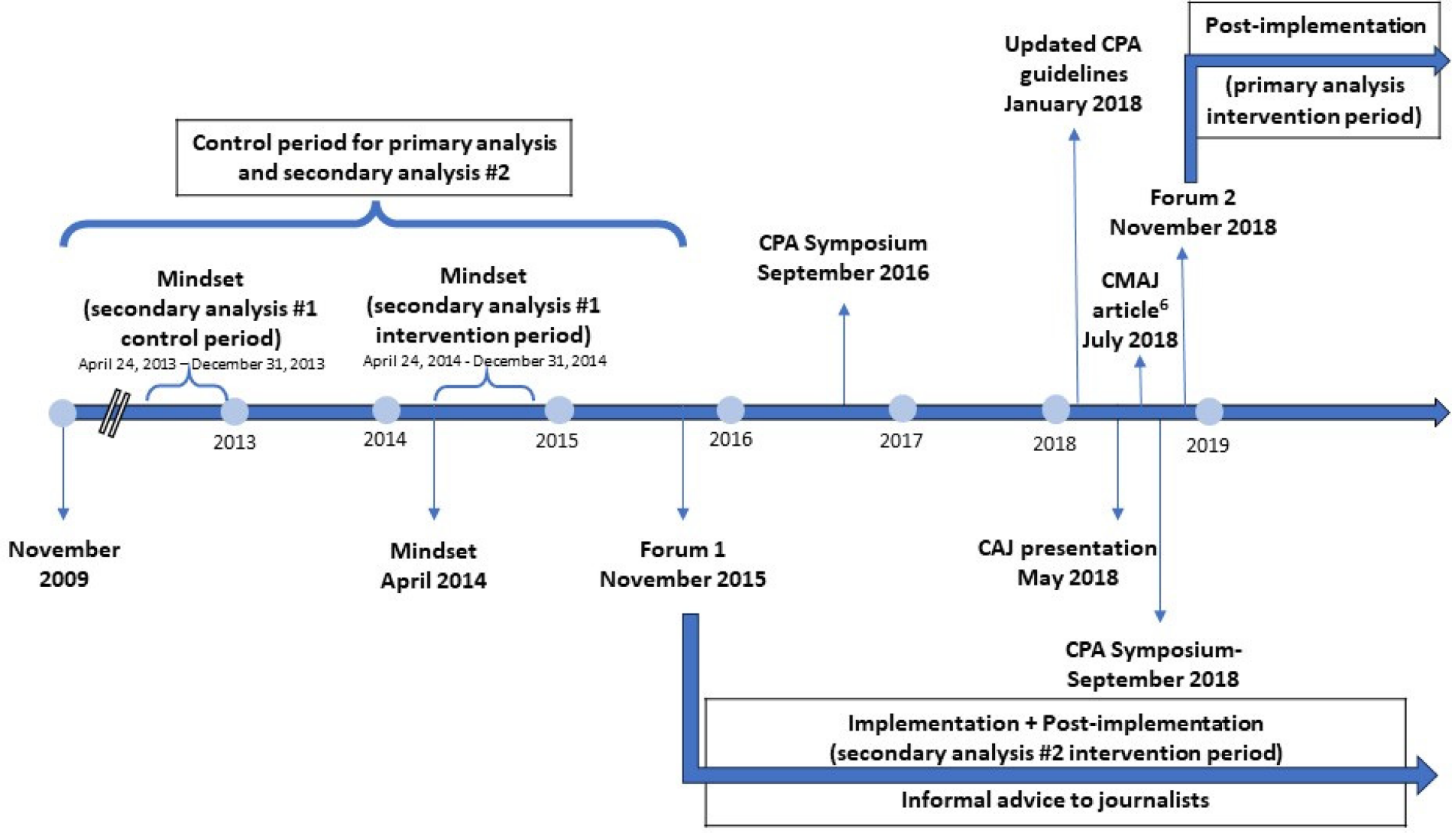
A timeline of Canadian national media engagement efforts regarding responsible reporting regarding suicide.

*Note*. CPA = Canadian Psychiatric Association; CAJ = Canadian Association of Journalists.

Responsible media reporting recommendations exist in Canada^19,20^ and worldwide.^21–24^ The World Health Organization (WHO) has listed “Interact with media on responsible reporting” as 1 of the 4 key evidence-informed strategies for population-level suicide prevention.^25^ A timeline of recent efforts in this area in Canada is presented in Figure 1. Canadian Journalists released their own “Mindset” guidelines for media reporting in April 2014, with a second edition released in 2017.^26^ Some suggestions contained within the initial edition of Mindset overlapped with those in international guidelines (e.g., communicate how people considering suicide can receive help and avoid reporting details of suicide methods). However, the message of the original Mindset guidelines differed substantially from expert guidelines. For example, Mindset suggested that imitative suicide following media reports does not occur,^26^ urged journalists not to “shy away from writing about suicide” and suggested they “cover suicides the same way they cover murders.”

In part, given concerns about some elements of Mindset, in November 2015, members of our group and Canadian collaborators launched a multipronged initiative to engage the Canadian national press. This included 2 fora with international experts discussing up-to-date scientific evidence with journalists, editors, and producers (November 2015 and November 2018),^27,28^ 2 symposia involving journalists at the Canadian Psychiatric Association (CPA) annual meeting (September 2016 and September 2018),^29,30^ the release and dissemination of updated CPA Guidelines for media reporting on suicide (January 2018),^19^ the first presentation on this topic by a suicide prevention expert at the Canadian Association of Journalists 2018 annual meeting (May 2018),^31^ and frequent and numerous other informal discussions with individual journalists. The impact of this national engagement strategy on reporting and suicide has yet to be investigated.

Given that the 2 initiatives, by our group and by journalists, overlapped substantially in terms of aims, timing, target audience, and content, it is practically challenging to distinguish between them and their public health impacts. Therefore, for this paper, we considered their impact together and heretofore refer to these combined efforts as “the national initiative” for improving suicide-related reporting (onset occurring on the date of the first joint forum, 6 November 2015).

We aimed to identify the potential impact of the national initiative on the content and overarching narrative of media reports and to test whether it was associated with fewer suicide deaths in the Greater Toronto Area (GTA). Our a priori hypotheses were that media reporting quality would improve in the postimplementation period following the initiative compared to the preinitiative period and that we would likewise observe fewer deaths by suicide postimplementation. Our secondary aims were (1) to identify any apparent immediate effects of the first edition of the Mindset guidelines (24 April 2014–31 December 2014) (secondary analysis 1), (2) to identify whether the onset of the combined initiative (6 November 2015) was associated with any improvements in reporting or reductions in suicides (secondary analysis 2), and (3) to characterize overarching narratives of reporting in the most recent years of available data.

## Methods

### Media Data

Media reports about suicide were the exposure of interest. We employed 2 media tracking companies (Meltwater and Dow Jones) using a previously developed list of suicide-related terms^6^ to search for relevant articles published in the top print and online media sources, by circulation, in the GTA market. The GTA was chosen as it is Canada's largest media market and the epicentre of the national engagement initiative and media outreach efforts. The specific publications comprised Canada's largest newspapers, its national public broadcaster, and the largest Toronto-based newspapers: *The Globe & Mail*, *National Post*, *Toronto Star*, *Toronto Sun*; online: CBC.ca, theglobeandmail.com, nationalpost.com, thestar.com. Articles with a major focus on suicide (including suicide death, attempts, and/or ideation), defined as suicide being the main or a major subject of the article, were included (“minor focus” articles with only a few sentences or a small paragraph about suicide were excluded).

Variables of interest were basic demographic and suicide-specific information about each article as well as the presence of a list of putatively harmful and/or putatively protective elements derived from responsible reporting guidelines for our prior study.^6^ Our prior study examined all major focus media reports for the years 2011–2014 and established good interrater reliability with respect to variables capturing general, putatively harmful and putative protective characteristics (note that, as we had all articles from 2011 to 2014 already coded, a random number generator was used to sample articles from those years).^6^ A new reliability check showed continued good agreement, with 1 exception; adequate reliability could not be re-established for the variable “statement of approval of suicide” and it was dropped from the analysis.

Given recent evidence of the importance of overarching narrative in suicide-related media,^18^ we also coded all articles in the most recent years of study (2020–2021) to characterize their “gestalt narrative.” A trained research assistant read titles and briefly screened all articles to identify their general thrust and/or message conveyed according to 7 potential narrative types that we constructed for this study: Death Attempt Stories (A-list Celebrity, B-list Celebrity, Villain, and Other), Survival Stories (A-list Celebrity, B-list Celebrity, Other, and Suicide Response), A Call for Action, Other Negative Message, Other Positive Message, Assisted Suicide/Medical Assistance in Dying (see Supplemental File for definitions and reliability testing results). An agreement was good for most variables, modest for “other positive message” and we were unable to establish an agreement for survival stories in A- or B-list celebrities as these articles were almost never present (there were none in the reliability test). These latter values should therefore be interpreted with a note of caution.

### Suicide Deaths

We collected coroner's data for those people determined to have died by suicide in the GTA (city of Toronto, Durham, Halton, Peel, and York regions) by the Office of the Chief Coroner of Ontario between 2009 and 2021 (6 years prior to and after implementation of the national initiative) using previously described methods.^32^

### Statistical Analyses

Due to the high volume of suicide-related articles, we employed a sampling approach to arrive at 900 articles (i.e., 75 articles/year) for the 12 years of the study. The 6-year preinitiative period was defined as November 2009–5 November 2015, inclusive. The 3-year implementation period, during which the major activities of the national initiative occurred, was defined as 6 November 2015–9 November 2018. The postimplementation period was defined as 10 November 2018–October 2021 inclusive.

The primary hypothesis that we would observe fewer putatively harmful and more putatively helpful elements following national initiative implementation (i.e., preinitiative vs. postimplementation period) was tested using Chi-square tests. Our secondary analyses aimed to identify: (1) the immediate effects of Mindset Guidelines before our group's outreach efforts began and (2) whether the onset of the combined initiative was associated with changes in reporting quality and/or suicides (preinitiative vs. implementation and postimplementation period). With respect to the latter, we used complete data for all suicide-related articles (2013–2014) from our original study^6^ to compare reporting content immediately after the release of the Mindset guidelines (24 April 2014–31 December 2014) to the same period in the prior year (24 April 2013–31 December 2013). Because an outlier event occurred during the exposure period (Robin Williams’ suicide), we conducted a sensitivity analysis in which articles about Williams were removed. Gestalt narratives for 2020–2021 articles are presented descriptively.

We tested the hypothesis of a reduction in suicide deaths associated with initiative onset with interrupted time-series analyses using the autoregressive integrated moving average (ARIMA) method as in previous studies^33^ to examine monthly suicide deaths in the GTA adjusting for autocorrelation and changes in population size, consumer price index and unemployment rate. In parallel with our media reporting quality analyses, we conducted a primary ARIMA analysis focused on changes in the postimplementation period and a secondary ARIMA analysis examining change across both the implementation and postimplementation periods. ARIMA model identification was done by considering the autocorrelation function and partial autocorrelation function. SPSS Expert Modeler was used to assist with model identification. Model fit was assessed with *R*-square (*R*^2^) and Ljung Box test was used to determine if the residuals or autocorrelations for the errors were nonzero. An ARIMA model (0,0,0)(0,0,1) was selected to model monthly suicide counts.

**Figure 2.**
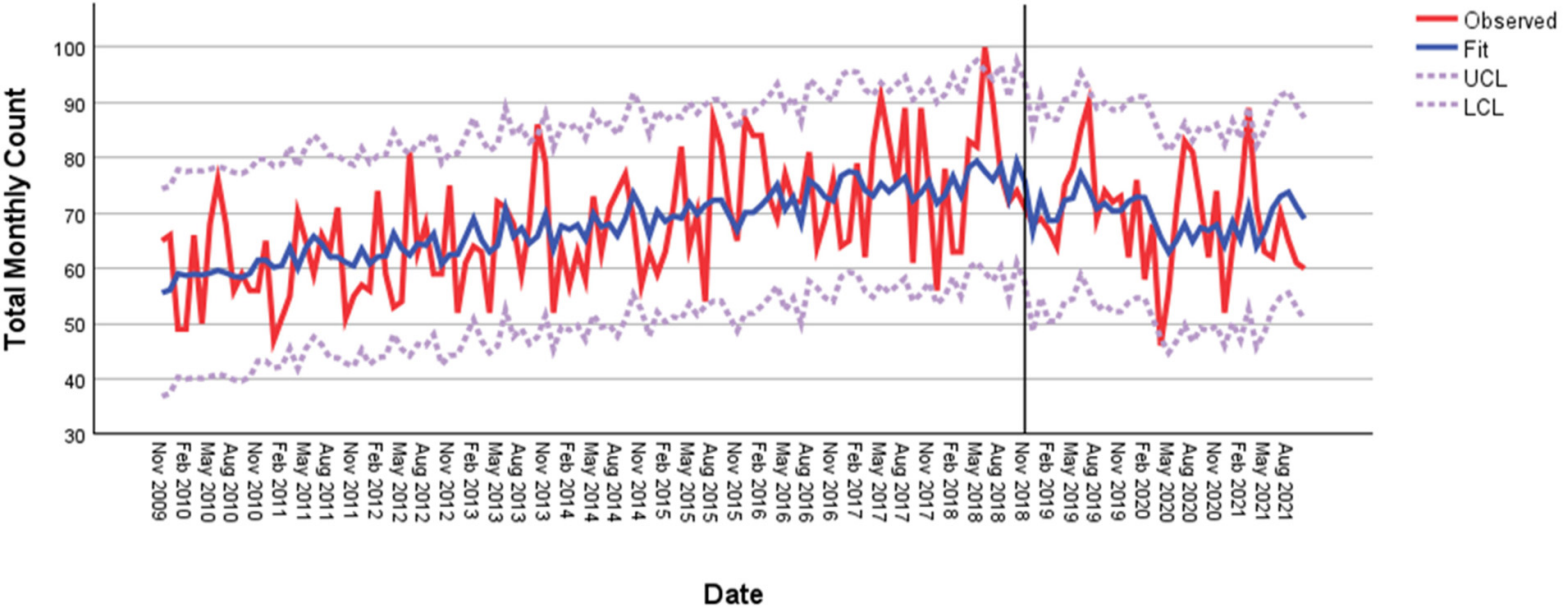
Monthly suicides in the GTA, November 2009–April 2021.

*Note*. The solid line represents the month by which the key implementation activities of the multipronged initiative to engage media had been completed. GTA = Greater Toronto Area; UCL = upper control limit; LCL = lower control limit.

All analyses were conducted using SPSS for Windows version 28.

## Results

### Media Reporting Quality

Demographic, suicide-specific, putatively harmful, and putatively protective elements of media articles for the preinitiative and postimplementation phases of the national initiative are presented in Tables 1 and 2 (see Supplemental File for a discussion of changes in general article characteristics). There were substantial differences in reporting between all periods.

**Table 1.** Characteristics of Articles Focusing on Suicide in Major Publications in Toronto Media Before and Following Implementation of a National Media Engagement Initiative (November 2009–5 November 2015 vs. 10 November 2018–October 2021).

Characteristics of media item	Total (%) *n* = 669	Preinitiative (%) *n* = 450	Postinitiative (%) *n* = 218	OR (95% CI)
**Item location**				
Print	415 (62.0)	280 (62.2)	135 (61.9)	1.00 (0.88 to 1.13)
**Suicide focus**				
Ideation	242 (36.2)	105 (23.3)	137 (62.8)	2.69 (2.21 to 3.28)
Attempt	192 (28.7)	82 (18.2)	110 (50.5)	2.77 (2.19 to 3.51)
Death	450 (67.3)	325 (72.2)	125 (57.3)	0.79 (0.70 to 0.90)
**Article focus**				
Specific person's death or suicidality	427 (63.8)	352 (78.2)	75 (34.4)	0.44 (0.36 to 0.53)
Suicide research	166 (24.8)	96 (21.3)	70 (32.1)	1.51 (1.16 to 1.96)
Suicide public policy	136 (20.3)	70 (15.6)	66 (30.3)	1.95 (1.45 to 2.61)
Assisted death	96 (14.3)	91 (20.2)	5 (2.3)	0.11 (0.05 to 0.28)
Individual murder-suicide	57 (8.5)	40 (8.9)	17 (7.8)	0.88 (0.51 to 1.51)
Mass murder-suicide	16 (2.4)	9 (2.0)	7 (3.2)	1.61 (0.61 to 4.25)
Suicide pact	11 (1.6)	11 (2.4)	0 (0)	-
Legal issues related to suicide	190 (28.4)	171 (38.0)	19 (8.7)	0.23 (0.15 to 0.36)
Suicide in fiction	24 (3.6)	20 (4.4)	4 (1.8)	0.41 (0.14 to 1.19)
**Article type**				
Opinion column	89 (13.3)	84 (18.7)	5 (2.3)	0.12 (0.05 to 0.30)
**Age focus**				
Youth	168 (25.1)	130 (28.9)	38 (17.4)	0.60 (0.44 to 0.83)
Adult	164 (24.5)	128 (28.4)	36 (16.5)	0.58 (0.42 to 0.81)
Older adults	45 (6.7)	40 (8.9)	5 (2.3)	0.26 (0.10 to 0.65)
**Gender focus**				
Male	345 (51.6)	243 (54.0)	102 (46.8)	0.87 (0.73 to 1.02)
Female	206 (30.8)	157 (34.9)	49 (22.5)	0.64 (0.49 to 0.85)

**Table 2.** Putatively Harmful and Protective Characteristics of Articles Focusing on Suicide in Toronto Media Before and Following Implementation of a National Media Engagement Initiative (November 2009–5 November 2015 vs. 10 November 2018–October 2021).

Characteristics of media item	Total (%) *n* = 669	Preinitiative (%) *n* = 450	Postinitiative (%) *n* = 218	OR (95% CI)
**Putatively harmful**				
Word “suicide” in the headline	165 (24.7)	122 (27.1)	43 (19.7)	0.73 (0.54 to 0.99)
Photo (deceased)	78 (11.7)	77 (17.1)	1 (0.5)	0.03 (0.00 to 0.19)
Photo (of someone looking sad)	15 (2.2)	14 (3.1)	1 (0.5)	0.15 (0.02 to 1.11)
Suicide method (in headline)	28 (4.2)	23 (5.1)	5 (2.3)	0.45 (0.17 to 1.16)
Suicide method (in text)	276 (41.3)	237 (52.7)	39 (17.9)	0.34 (0.25 to 0.46)
Method described in detail	91 (13.6)	54 (12.0)	37 (17.0)	1.41 (0.96 to 2.08)
Favourable characteristic (deceased)	50 (7.5)	44 (9.8)	6 (2.8)	0.28 (0.12 to 0.65)
Statement that suicide is inevitable	1 (0.1)	1 (0.2)	0 (0)	—
Sensationalistic reporting	13 (1.9)	10 (2.2)	3 (1.4)	0.62 (0.17 to 2.23)
Glorified or romanticized suicide	6 (0.9)	4 (0.9)	2 (0.9)	1.03 (0.19 to 5.59)
Reasons for suicide (simplistic)	84 (12.6)	39 (8.7)	45 (20.6)	2.38 (1.60 to 3.54)
Identifies deceased as a celebrity	51 (7.6)	41 (9.1)	10 (4.6)	0.50 (0.26 to 0.99)
Interview with the bereaved*	113 (16.9)	88 (19.6)	25 (11.5)	0.59 (0.39 to 0.89)
**Putatively protective**				
Unfavourable characteristic (deceased)	8 (1.2)	6 (1.3)	2 (0.9)	0.69 (0.14 to 3.38)
Alternatives to suicide	93 (13.9)	57 (12.7)	36 (16.5)	1.30 (0.89 to 1.92)
Community resources	66 (9.9)	5 (1.1)	61 (28.0)	25.2 (10.3 to 61.8)
Positive outcome of a suicide-related crisis	19 (2.8)	4 (0.9)	15 (6.9)	7.74 (2.60 to 23.05)
Warning signs of suicidal behaviour	80 (12.0)	4 (0.9)	76 (34.9)	39.2 (14.5 to 105.8)
How to approach someone	49 (7.3)	1 (0.2)	48 (22.0)	99.1 (13.8 to 713.1)
Message of hope	43 (6.4)	19 (4.2)	24 (11.0)	2.61 (1.46 to 4.66)

*Guidelines urge caution in reporting interviews with the bereaved but this may not necessarily be harmful.

### Primary Analysis: Pre–Post Differences Following Initiative Implementation

#### Putatively Harmful Content

The national initiative was associated with reductions in the proportion of articles with suicide in the headline (27.1% pre vs. 19.7% post; OR 0.73, 95% CI, 0.54 to 0.99), presenting photos of a person who had died by suicide (17.1% pre vs. 0.5% post; OR 0.03, 95% CI, 0.00 to 0.19), and emphasizing favourable characteristics of someone who had died by suicide (9.8% pre vs. 2.8% post; OR 0.28, 95% CI, 0.12 to 0.65). We also observed a large reduction in the proportion of articles mentioning the suicide method in the article text (52.7% pre vs. 17.9% post; OR 0.34, 95% CI, 0.25 to 0.46). Following the national initiative, a significantly smaller proportion of articles reported on suicide in celebrities (9.1% pre vs. 4.6% post; OR 0.50, 95% CI, 0.26 to 0.99) or included interviews with those who were bereaved by suicide (19.6% pre vs. 11.5% post; OR 0.59, 95% CI, 0.39 to 0.89). However, 1 putatively harmful characteristic, simplistic reasons for suicide, was more common after the initiative (8.7% pre vs. 20.6% post; OR 2.38, 95% CI, 1.60 to 3.54).

#### Putatively Protective Content

All of the putatively protective content encouraged by guidelines increased following the commencement of the national initiative, with some large increases (community resources: 1.1% pre vs. 28.0% post; OR 25.2, 95% CI, 10.3 to 61.8; warning signs: 0.9% pre vs. 34.9% post; OR 39.2, 95% CI, 14.5 to 105.8); how to approach someone: 0.2% pre vs. 22.0% post; OR 99.1, 95% CI, 13.8 to 713.1), some moderately sized increases (positive outcome of a suicidal crisis: 0.9% pre vs. 6.9% post; OR 7.74, 95% CI, 2.60 to 23.1; messages of hope: 4.2% pre vs. 11.0% post; OR 2.61, 95% CI, 1.46 to 4.66), and 1 nonsignificant trend observed (alternatives to suicide: 12.7% pre vs. 16.5% post; OR 1.30, 95% CI, 0.89 to 1.92).

### Suicide Deaths

The fitted model with observed monthly suicide deaths is shown in Figure 2 (Ljung Box: no significant autocorrelations; model displayed good fit: *Q*  =  20.74; *df*  =  17; *p*  =  0.24). The model explained 29% of the variation in monthly suicide deaths in the GTA. Mean monthly suicide deaths for the postimplementation period, after controlling for potential confounders, were 62.2  ±  15.9 compared to 70.1  ±  11.9 before this period (relative risk [RR] 0.89, 95% CI, 0.82 to 0.97). The impact of this decrease in the number of suicide deaths in the postimplementation period was not significant (*ω* = −5.41, *SE*  =  3.43, *t*  =  1.58, *p*  =  0.12). Population size and consumer price index were nonsignificant covariates; however, the unemployment rate was significant (*ω* = −1.55, *SE*  =  0.64, *t*  =  −2.44, *p*  =  0.02).

### Immediate Impact of Mindset Guidelines (Secondary Analysis 1)

Changes following the release of Canadian journalists’ own Mindset Guidelines 18 months earlier are discussed in the Supplemental File and presented in Supplemental Tables S1 and S2. In short, no reductions in harmful content were observed immediately following the publication of the Mindset Guidelines with increases in method in detail, suicide inevitable, simplistic reasons, and sensationalistic reporting as well as alternatives to suicide and warning signs of suicidal behaviour.

### Pre–Post Differences From Initiative Onset (Secondary Analysis 2)

Findings of our secondary analysis including the implementation phase of the initiative were largely consistent with the overall study findings for putatively harmful and protective content (see Supplemental Results and Supplemental Tables S5 and S6). Monthly suicide deaths were also unchanged (see Supplemental Figure S1).

### Gestalt Narratives

We identified 3,065 articles with a major focus on suicide published during the most recent years for which we had data (2020–2021; Table 3). The most common “Gestalt” narrative was a story emphasizing a suicide death or attempt (55%), followed by stories with an Other Negative Message (21%). Fewer than 4% of all articles emphasized a story of survival and only 1% of articles emphasized an Other Positive Message.

**Table 3.** Gestalt Narratives of Articles With a Major Focus on Suicide in the Toronto Media Market 2020–2021 (*n*  =  3,065).

Narrative type		Articles count	Percentage
Death attempt stories	A-list celebrity	40	1.31%
	B-list celebrity	95	3.10%
	Villain	809	26.39%
	Other	749	24.44%
Survival stories	A-list celebrity	9	0.29%
	B-list celebrity	22	0.72%
	Other	79	2.58%
Suicide response		179	5.84%
A call for action		219	7.15%
Other negative message		638	20.82%
Other positive message		35	1.14%
Assisted suicide/medical assistance in dying		191	6.23%

## Discussion

Our study aimed to characterize the impact of a national media engagement initiative to improve the quality of suicide-related reporting in Canada. We found that, postimplementation, the national initiative was associated with substantial reductions in putatively harmful content and across-the-board increases in the proportion of articles with putatively protective content, a finding aligned with other recent Canadian research on media and suicide.^34–36^ This finding differs somewhat from what we observed immediately after the release of the Mindset Guidelines and following the combined initiative onset. The release and dissemination of Mindset Guidelines coincided with some positive changes that appeared to persist over time (e.g., a greater proportion of articles presenting alternatives to suicide), but also negative changes that persisted for some years (e.g., a greater proportion of articles describing suicide methods in detail, presenting simplistic reasons for suicide). The implementation phase of the national initiative was also associated with increases in putatively harmful content which did not persist into the postimplementation period (suicide in the headline, suicide presented as inevitable).

Of note, Mindset Guidelines themselves have had 3 iterations over the past decade and have discarded some of their more problematic original aspects. For example, whereas the original Mindset Guideline dismissed concerns about suicide contagion as not “borne out in the long run by independent statistics,” the most recent edition emphasizes concerns about contagion effects as follows:

Contagion—in which learning of one person's death may prompt other desperate people to [die by suicide]—is a clinical concern supported by robust evidence, particularly when the initial death is that of a celebrity… Clearly these are circumstances in which journalists should try hard to minimize harm.^37^

Indeed, in the context of the national initiative, a primary author of the original Mindset Guidelines joined the authorship team of the updated CPA Guidelines for media reporting on suicide.^19^ Likewise, members of the national engagement initiative group provided input into the most recent iteration of Mindset. We speculate that this collaboration is likely to have had a substantial impact on the results of our study. That is, efforts in Canada began as 2 competing initiatives 1 of which (Mindset) included at least some encouragement of journalists to engage in reporting that did not align with international best practices. For some years after, it is understandable that results were mixed. Following dialogue and consultation, the 2 simultaneous efforts had a more consistent message. By late 2018, it was no longer possible to practically disentangle the 2, and our unified initiative had a more consistently positive impact. These findings are encouraging and underscore the importance of ongoing engagement between scientific experts and journalists.

Our results related to suicide deaths also appear notable. We found weak evidence of a reduction in suicides in the *postimplementation period* that was not observed when we focused on the *onset* of the national initiative as an inflection point. Prior research from Austria and Germany showed that educating journalists about the potential harms of suicide-related media reports was associated with improved reporting and fewer suicides.^38,39^ Australia's media guidelines were estimated to prevent more than 100 suicides over a 5-year period and appeared highly cost-effective.^40^ Guidelines had been disseminated to the Austrian press since 1987, and the original study that coined the Papageno effect showed that they presented Papageno narratives in 9% of their suicide-related articles in 2005.^14^ In contrast, in 2020–2021, after several years of our media engagement initiative which placed a heavy emphasis on encouraging Papageno narratives, the Canadian press reported on such narratives in fewer than 4% of articles.

In this context, our results largely comport with expectations. As described above, the implementation phase of the initiative was characterized by some mixed messages towards journalists, a mixed impact on reporting quality, and no decrease in suicide counts. Once messaging of the joint initiative was brought into better alignment, reporting was more clearly improved, and there was a nonsignificant trend towards fewer suicides. There may be multiple explanations for that finding, but 1 important potential contributor was the Canadian media's continued emphasis on negative suicide-related Gestalt narratives. These were unlikely to leave those exposed with the impression that people can survive and overcome suicidal crises. Under such circumstances, it would be hard to envision a scenario whereby reporting would lead to substantial reductions in suicide deaths. This is because stories of hope and recovery appear to be the “active ingredient” in preventing suicide in some at-risk individuals.^2^ This conclusion arises from a confluence of data including research showing that dissemination of a popular hip-hop song about survival and tweets about suicide prevention were each associated with more subsequent calls to the US national helpline and fewer suicides.^41–43^ Our group recently published studies designed to examine the overarching narrative of stories, including 1 specifically focused on Canada; these indicated that different narratives were associated with expected subsequent changes in suicide rates.^18,43^

Although the initiative was not associated with a demonstrable reduction in suicide deaths, media guidelines and journalist engagement remain of crucial importance; these efforts must account for the perspectives of journalists^27,44,45^ and, ideally, dissemination would begin in journalism schools (e.g., guidelines are included in all journalism school curricula in Australia). These efforts and curricula must be updated over time to include the latest research evidence. Currently, this includes the importance of dissemination of narratives of survival.

Prior research has demonstrated that dissemination of media guidelines specifically can lead to improved reporting; however, effects can be variable and may fluctuate over time and, often, journalist awareness and use of guidelines can be low, particularly in low- and middle-income countries.^32,46–66^ The discrepancy between our encouraging putatively harmful/protective element outcomes and our stark Gestalt narrative findings suggest that the current lists of “dos” and “don’ts” provided to journalists may not only miss the forest for the trees, but indeed leave suicide-related media reporting in the wrong forest altogether. It is worth underscoring that societal messaging and narratives about suicide and its prevention appear to differ from public messaging about almost any other health outcome. Consider how the public would react if 63% of pandemic-related articles focused on the story of a COVID-19 death with only 11% conveying a message of hope that survival is possible with vaccines. Such findings would be of great concern, yet these are equivalent to our *improved* suicide-related reporting findings *after* the national initiative was implemented.

This study has several limitations. As with other population-level studies of media reporting, we were unable to determine the degree to which people who died by suicide in the GTA were or were not exposed to the media reporting studied. Further, it is uncertain whether our results are generalizable to the rest of Canada. Specifically, the national initiative was mainly directed at Canada's national media (i.e., the major press broadly circulated across the country) and our analysis focused on the content of reporting by this group. The degree to which the national initiative may or may not have had an impact on regional outlets including French-language outlets (e.g., much of the media in Quebec) is unknown. We must emphasize that other efforts to change discourse outside the national initiative, for example, Bell Canada's Let's Talk campaign, may have played a role; although there is some evidence that such initiatives have not, as yet, improved suicide-related discourse.^67^ Importantly, the degree to which each component of the national initiative, the Mindset Guidelines, and/or unrelated factors (e.g., evolving societal beliefs about suicide) may have contributed to our findings is unknown. Our results do suggest the possibility that the national initiative and Mindset may have worked synergistically in certain respects but also that some of the original Mindset recommendations might have undermined the message of the national initiative. Our study also does not account for the fact that Canadians increasingly consume information and news using social media,^68^ and also the fact that they may be influenced by the entertainment media.^69^ Results of our Gestalt narrative findings may also have been influenced by the fact that these years spanned the COVID-19 pandemic and it is unclear if they may be generalizable to other epochs.^70^ Lastly, this was an exploratory study that did not correct for multiple testing and therefore the results related to specific variables should be interpreted with caution and require replication.

Our study found that a national media engagement initiative to improve suicide-related reporting had beneficial impacts on news article content but that the overarching narratives remained focused on death rather than survival. There was no evidence that the initiative impacted suicide rates. These findings underscore what may be a fundamental conceptual problem with existing approaches to media engagement and point to the need for an increased emphasis on the importance of narrative.

## Supplemental Material

sj-docx-1-cpa-10.1177_07067437231223334 - Supplemental material for Changes in Media Reporting Quality and Suicides Following National Media Engagement on Responsible Reporting of Suicide in Canada Supplemental material, sj-docx-1-cpa-10.1177_07067437231223334 for Changes in Media Reporting Quality and Suicides Following National Media Engagement on Responsible Reporting of Suicide in Canada by Mark Sinyor, MSc, MD, FRCPC, Daniella Ekstein, MA, Nivetha Prabaharan, MA, MSW, Lisa Fiksenbaum, PhD, Caroline Vandermeer, Ayal Schaffer, MD, FRCPC, Jane Pirkis, PhD, Marnin J. Heisel, PhD, C.Psych., Benjamin I. Goldstein, MD, PhD, FRCPC, Donald A. Redelmeier, MD, MSHSR, FRCPC, FACP, Paul Taylor and Thomas Niederkrotenthaler, MD, PhD in The Canadian Journal of Psychiatry
