# Supplementary material for: Changes in Media Reporting Quality and Suicides Following National Media Engagement on Responsible Reporting of Suicide in Canada: Changements de la Qualité des reportages dans les médias sur les suicides suite à l’engagement des médias nationaux à la déclaration responsable du suicide au Canada
Source: Can J Psychiatry. 2024 Jan 4;69(5):358–68. doi: 10.1177/07067437231223334 (PMC11032096; doi:10.1177/07067437231223334)
Supplement: sj-docx-1-cpa-10.1177_07067437231223334 - Supplemental material for Changes in Media Reporting Quality and Suicides Following National Media Engagement on Responsible Reporting of Suicide in Canada [file sj-docx-1-cpa-10.1177_07067437231223334.docx]

Sunnybrook Research Institute - Suicide Media Search Initiative

**Free text search:**

suicide OR suicidal OR killed himself OR killed herself OR kill himself OR kill herself OR hung himself OR hung herself OR took his life OR took her life OR take his life OR take her life OR end his own life OR end her own life OR ended his own life OR ended her own life OR end his life OR end her life OR ended his life OR ended her life OR ends his life OR ends her life

**Date:**

July 2016 – December 2021

**Duplicates:**

Include duplicates.

**Sources:**

From Meltwater:

- *Print:* Maclean’s Magazine, National Post, Toronto Sun, 24 Hours Toronto
- *Online:* CBC.ca, nationalpost.com

From Factiva:

- *Print:* The Globe & Mail, Toronto Star
- *Online:* theglobeandmail.com, thestar.com

**Language:**

English

**Sunnybrook Research Institute – Suicide Media Data Dictionary**

| **TITLE** | **DESCRIPTION** | **CODING/EXAMPLE** |
| --- | --- | --- |
| Date | Publication date | yy-dd-mm |
| Publication Name | Name of the journal/magazine/website in which the article appears | i.e. The Globe and Mail, National Post, thestar.com, nationalpost.com, etc |
| **SPECIAL ITEM TYPE** | | |
| Opinion Piece | Listed as an editorial article (including letters to the editor) or when item author overtly discusses a personal opinion. | 0 = No, 1 = Yes  i.e. an editorial article |
| Advice Column |  | 0 = No, 1 = Yes |
| Fictional | The item discusses a work of fiction, fictional portrayal, or the piece itself is fiction. | 0 = No, 1 = Yes, 2 = Both actual and fictional events are discussed |
| **ITEM MENTIONS** | | |
| Suicidal Thoughts | Talks about how often people think about suicide or a person’s experience contemplating suicide (allusions are insufficient). | 0 = No, 1 = Yes |
| Suicide Attempt | Talks about a specific suicide attempt(s) or suicide attempt statistics. Self-mutilation or self-harm will not be coded. Articles that only state someone has been placed on suicide watch or declared a suicide risk will not be coded. | 0 = No, 1 = Yes  i.e. “8% of young people attempt suicide..” |
| Suicide Death | Talks about a specific suicide death(s) or suicide death statistics. | 0 = No, 1 = Yes |
| **FOCUS ITEMS** | | |
| Individuals Experience | Describes a specific person’s/multiple people’s experience with suicidal thoughts/attempt/death. | 0 = No, 1 = Yes |
| Suicide Research | The item describes suicide research or statistics about suicide. | 0 = No, 1 = Yes  i.e. “Suicide is contagious among young people, new Canadian research shows…” |
| Suicide Policy Programs | The publication describes policy measures, programs or suggestions designed to prevent suicide such as policies that prevent bullying, putting barriers up on bridges, new ways for institutions to assess suicide risks, or even a Canada-wide suicide prevention plan. | 0 = No, 1 = Yes |
| Institutional Protest | The item discusses suicidal thoughts (including threats), attempts, or deaths as related to a protest towards a given institution. | 0 = No, 1 = Yes  i.e. Hunger strikes, self-immolations, etc. |
| Assisted Suicide | The item discusses assisted suicide. | 0 = No, 1 = Yes |
| Murder Suicide: Individual | The item describes a murder suicide. This category should also be used for attempted murder suicide such as an article where a student shot other students who, for example, were sent to hospital and he then killed himself. | 0 = No, 1 = Yes  i.e. “The mother of a 3-year-old thrown to his death from a 52-story apartment building said the father killed the boy - and then himself…” |
| Murder Suicide: Mass | The item describes a mass murder suicide. Generally quantified as more than six people who were not known to the victim (meaning that if a large family was killed, it would be coded as an individual murder). | 0 = No, 1 = Yes |
| Multiple Suicide: Pact | The item describes a suicide pact. | 0 = No, 1 = Yes |
| Focus Legal Issues | The item describes legal issues related to suicide such as a lawsuit or complaint arising from someone’s suicide death or a law being implemented in order to prevent suicide or its causes (e.g. a law to stop bulling or a gun law designed to prevent mentally ill people from having access to guns) | 0 = No, 1 = Yes |
| **ITEM AGE FOCUS**  ***These will be coded so long as one can assume without reasonable doubt that a person falls in a given age range** | | |
| Major: Teen Youth | If the publication’s content has a large focus on the <25 age group. | 0 = No, 1 = Yes |
| Major: Adult | If the publication’s content has a large focus on the 25-65 age group. | 0 = No, 1 = Yes |
| Major: Elderly | If the publication’s content has a large focus on the >65 age group | 0 = No, 1 = Yes |
| **GENDER FOCUS** | | |
| Male/ Female | Does the article mention suicidal thoughts/attempts/deaths in boys/males or girls/females or in a specific man or woman. Multiple responses allowed. | Male 0 = No, 1 = Yes  Female 0 = No, 1 = Yes  An article stating “2,000 men died if suicide in Canada in 2011” and “Jane Smith took her life on September 1^st^” should be coded as both male and female. |
| **CONTENT ITEMS** | | |
| Photo Picture of the **Deceased** | A photo of the **deceased** accompanies the article/publication. Only use for deceased – do not code if a picture of someone who has thought of or attempted suicide. We will google each article to verify whether a photo accompanied the story. | 0 = No, 1 = Yes |
| Photo Picture of Someone Looking Sad | A photo of a person or people looking sad/somber accompanies the article/publication. We will google each article to verify whether a photo accompanied the story. Articles of mass murders where the photographed people are not grieving the person who committed suicide have not been coded. | 0 = No, 1 = Yes |
| Mention of Suicide Method in Headline | The article’s headline specifically identifies the method of suicide. It does not necessarily need to have the word ‘suicide’. Shootings are not coded as in the headline unless they mention the suicide in the headline as well (ex. “Alberta highway shooting that took four lives; Gunman among the dead” and “Gun control keeps suicides down” would be coded 1, “Gunman in Alberta highway shooting had three loaded weapons” and “Firearms by the numbers” would be 0, as the attempt/death is not mentioned.) | 0 = No, 1 = Yes  i.e. “Despite attempted intervention by police, man found hanging…” |
| **MENTIONS OF METHOD** | | |
| Mention of Suicide Method in Text |  | 0 = No, 1 = Yes |
| Described Hanging |  | 0 = No, 1 = Yes |
| Described Self-Poisoning Overdose |  | 0 = No, 1 = Yes |
| Described Jumping Building |  | 0 = No, 1 = Yes |
| Described Jumping Bridge |  | 0 = No, 1 = Yes |
| Described Railway Death | Includes all incidents of jumping in front of trains. | 0 = No, 1 = Yes |
| Described Car Exhaust |  | 0 = No, 1 = Yes |
| Described Other Asphyxia | Includes helium, suffocation, etc. | 0 = No, 1 = Yes |
| Described Firearm Shooting |  | 0 = No, 1 = Yes |
| Described Suicide by Cop |  | 0 = No, 1 = Yes |
| Described Cutting Stabbing |  | 0 = No, 1 = Yes |
| Described Drowning |  | 0 = No, 1 = Yes |
| Euthanasia / Lethal Injection | Not coded if it only says “doctor-assisted” | 0 = No, 1 = Yes |
| Described Burning / Electrocution |  | 0 = No, 1 = Yes |
| Described Other | The publication specifically describes that the method used in a/the suicide was with the use of some other method not already covered in previous variables | 0 = No, 1 = Yes |
| If Method Described: Described In Detail | Will be coded so long as the process of their suicide has been described (sequence of events). Articles which include any additional details to the method were also coded. I.e. “He hanged himself with a tie” would be coded as in detail versus, “He hanged himself” which would not. | 0 = No, 1 = Yes  i.e. "person X researched ways to die, went to a store, bought a gun and shot himself" |
| **VALUE JUDGEMENT OF THE DECEASED**  ***Will not include situational descriptions such as a positive/negative emotional experiences** | | |
| Unfavourable Characteristic |  | 0 = No, 1 = Yes  i.e. “He was a terrible man…” “He was evil…”, “He never did anything right” |
| Favourable Characteristic |  | 0 = No, 1 = Yes  i.e. “He was a wonderful person…” or “She was gentle…” |
| **ARTICLE CONTENT** | | |
| A Statement that Suicide is Inevitable |  | 0 = No, 1 = Yes  i.e. “…there was no way to stop it…” or “it was going to happen no matter what anyone did…” |
| Sensationalistic Reporting | Written in a tabloid manner to excite the reader. | 0 = No, 1 = Yes |
| Glorified Romanticized reasons for the Suicide | The rationale for the suicide is portrayed as noble and the person is seen as a hero. | 0 = No, 1 = Yes  i.e. "she did it to be with her children" “he was a hero to those who watched him on the internet” |
| Simplistic reasons for the Suicide | A single step cause and effect or the suicide is portrayed as ‘merely a social phenomenon’ as opposed to being ‘related to a mental disorder’. Please note that if the article is written to dispel this idea (e.g. Margaret Wente Oct 29, 2013) then this should not be coded. | 0 = No, 1 = Yes  i.e. a single step cause and effect - e.g. "he committed suicide because he was angry at his mother" or "she jump because she had lost her job"  i.e For assisted suicide articles: “to end the pain” or “die gracefully” |
| A Statement of Approval of the Suicide | Either the author of the article or someone described within the piece agreed with or encouraged the person’s suicide. Must apply to a specific person’s death. For example, editorial columns where readers agree with the notion of assisted suicide should not be coded as approval of suicide unless they are agreeing with a specific person’s death by assisted suicide. | 0 = No, 1 = Yes  i.e. “…many encouraged him and suggested alternative ways to take his life” |
| Item notes the deceased was a Celebrity | This would apply to anyone who was famous prior to their suicide death but not because of it (Would include Robin Williams but not Amanda Todd). Should also be coded for a celebrity with suicidal thoughts or suicide attempts. | 0 = No, 1 = Yes |
| Item includes interview with the Bereaved | Very brief mentions will be included. | 0 = No, 1 = Yes |
| Item includes alternatives to suicide such as Seeking Treatment | This should be explicit. It is not enough to say that “we interviewed Dr. X from hospital Y which treats depression”. It should be a statement like “getting help for depression may decrease suicidal thoughts” – even if these are listed as not effective. For assisted suicide articles, palliative care has been included as an alternative. | 0 = No, 1 = Yes |
| Item includes community resource information for those with suicidal ideation | Article specifically tells the reader where they can go or who they can call in order to get help. | 0 = No, 1 = Yes |
| Item includes examples of a positive outcome of suicidal crisis |  | 0 = No, 1 = Yes  i.e. someone calling a suicide hotline/getting help |
| Item includes warning signs of suicidal behaviour | This should be explicit. Item includes text specifically indicating some warning signs of suicide or a link to materials that outline them. | 0 = No, 1 = Yes  i.e. “Warning signs of suicide include…” or “Get help if your teen experiences…” |
| Item includes how to approach a suicidal person |  | 0 = No, 1 = Yes |
| Item includes a message of hope that suicidal ideation and/or behaviour is treatable | Item includes a clear statement of hope that suicidal thoughts/behaviour can get better or be overcome. | 0 = No, 1 = Yes |

Gestalt Narrative Definitions

Death/attempt story: A story of an individual or small number of individuals who attempt suicide or end their lives.

Survival story: A story of an individual or small number of individuals who contemplate suicide/are at risk of suicide but then seek help/find hope/survival.

Suicide response: An article about attempts/deaths in individuals and groups but the emphasis is on doing something to help (e.g. preventing bullying, funding suicide prevention initiatives).

A call for action: An article with a negative message providing information about attempts/deaths and calling for attention and resources (but no initiative is actually taking place yet).

Other negative message: An article with some ominous/unhappy message about suicide (e.g. how football concussions are causing players to contemplate suicide) not fitting into the above categories.

Other positive message: An article with an uplifting message about suicide/suicide prevention not fitting into the above categories.

Assisted Suicide/Medical Assistance in Dying: Article with an emphasis on physician assisted death.

Notes:

A-list celebrity: Someone likely known by the average person in the population under study

B-list celebrity: Someone likely known by a subset of the population under study

Villain: Someone with notoriety for wrongdoing (e.g. criminal acts)

Other: Someone not fitting into any of the above categories

Reliability testing for Gestalt Narrative codes:

| **Gestalt Narrative Type** | | **Cohen’s Kappa** |
| --- | --- | --- |
| Death Attempt Stories | A-list Celebrity | 0.920454545 |
|  | B-list Celebrity | 1 |
|  | Villain | 0.91047503 |
|  | Other | 0.860079954 |
| Survival Stories | A-list Celebrity | undefined* |
|  | B-list Celebrity | undefined* |
|  | Other | 1 |
| Suicide Response | | 0.90648855 |
| A Call for Action | | 0.753954306 |
| Other Negative Message | | 0.791108404 |
| Other Positive Message | | 0.589958159 |
| Assisted Suicide/Medical Assistance in Dying | | 0.938845554 |

Supplementary Results

Sensitivity Analysis (Mindset)

See Supplementary Tables 1-4. The Mindset sensitivity analysis excluding articles about Robin Williams yielded similar results, with differences largely indicative of the specific details of his death. That is, when articles about Williams were included, there were apparent increases in articles about males, celebrities, that described the deceased in favourable terms, and that included a photo of the deceased. These were not observed when articles about Williams were excluded.

General Article Characteristics – Primary Analysis

Following the national initiative, a greater proportion of articles told the story of people experiencing suicidal thoughts (23.3% pre vs. 62.8% post; OR 2.69, 95%CI 2.21-3.28) and suicide attempts (18.2% pre vs. 50.5% post; OR 2.77, 95%CI 2.19-3.51) and a smaller proportion reported on suicide deaths (72.2% pre vs. 57.3% post; OR 0.79, 95%CI 0.70-0.90).

There was also a large shift away from articles about specific individual’s experiences related to suicide (78.2% pre vs. 34.4% post; OR 0.44, 95%CI 0.36-0.53) and legal issues related to suicide (38.0% pre vs. 8.7% post; OR 0.23, 95%CI 0.15-0.36) with more articles related to research (21.3% pre vs. 32.1% post; OR 1.51, 95%CI 1.16-1.96) and public policy (15.6% pre vs. 30.3% post; OR 1.95, 95%CI 1.45-2.61).

In keeping with the decreased emphasis on stories about individuals after the national initiative commenced, a smaller proportion of articles focused on specific demographic groups including youth (28.9% pre vs. 17.4% post; OR 0.60, 95%CI 0.44-0.83), adults (28.4% pre vs. 16.5% post; OR 0.58, 95%CI 0.42-0.81), older adults (8.9% pre vs. 2.3% post; OR 0.26, 95%CI 0.10-0.65), and women (34.9% pre vs. 22.5% post; OR 0.64, 95%CI 0.49-0.85).

There were also far fewer articles on “assisted suicide” after the launch of the national initiative (20.2% pre vs. 2.3% post; OR 0.11, 95%CI 0.05-0.28), although this is a potentially misleading finding that almost certainly owes to a nomenclature change (around the time the national initiative began, “assisted suicide” was rebranded in Canada as “Medical Assistance in Dying” and our search was not designed to capture that term).

General Article Characteristics – Secondary Analysis Focused on Pre-Initiative vs. Implementation and Post-Implementation Phase

The analysis in which we included articles from the implementation phase largely yielded similar results to the main analysis. With respect to general article characteristics, there were now fewer print articles over time, no finding of fewer articles about youth suicide, and a trend toward a reduction in articles about males became significant.

Immediate Impact of Mindset Guidelines (Secondary Analysis #1)

No reductions in harmful content were observed immediately following publication of the Mindset Guidelines; three of the observed increases in harmful content began following their publication (method in detail:8.8% pre vs. 11.9% post; OR 1.40, 95%CI 1.08-1.82; suicide inevitable: 0.2% pre vs. 1.0% post; OR 4.20, 95%CI 1.18-14.95; simplistic reasons:4.1% pre vs. 15.3% post; OR 4.24, 95%CI 3.07-5.85). Release of the Mindset guidelines was also associated with an increase in sensationalistic reporting with a tabloid framing (1.8% pre vs. 4.3% post; OR 2.48, 95%CI 1.49-4.12) that did not persist over time.

Release of the Mindset Guidelines was associated with a substantial increase in the proportion of articles with alternatives to suicide (15.8% pre vs. 28.7% post; OR 2.14, 95%CI 1.76-2.60) and a small absolute increase in the proportion of articles describing warning signs of suicidal behaviour (0.1% pre vs. 1.3% post; OR 15.85, 95%CI 2.09-120.18).

The trend toward a smaller proportion of articles on specific individuals’ experiences and a greater proportion related to research was already observed following the release of the Mindset Guidelines (individual experiences: 82.5% pre vs. 76.3% post; OR 0.68, 95%CI 0.56-0.83; research 15.5% pre vs. 23.1% post; OR 1.63, 95%CI 1.33-2.00). Reductions in proportions of articles about females were already apparent after release of the Mindset Guidelines (45.5% pre vs. 36.6% post; OR 0.68, 95%CI 0.57-0.80) and their release was also associated with a smaller proportion of articles about youth that did not appear to persist over time (33.4% pre vs. 24.3% post; OR 0.63, 95%CI 0.53-0.76).

Results of the sensitivity analysis excluding articles about Robin Williams’s suicideResults of the sensitivity analysis were unremarkable (see Supplementary Tables 3 and 4) (see Supplementary File).

Impact of The National Initiative from Initiative Onset (Secondary Analysis #2)

After onset of the national initiative, there were fewer suicide-related articles that included a photo of someone looking sad (3.1% pre vs. 0.9% post; OR 0.29, 95%CI 0.09-0.86). Post-hoc testing also identified decreased mentions of almost all suicide methods after onset of the initiative (Supplementary Table 7).

We again found a higher proportion of articles described simplistic reasons for suicide after onset of the national initiative (8.7% pre vs. 22.4% post; OR 2.59, 95%CI 1.83-3.65). Notably, several other types of putatively harmful content also occurred more frequently after national initiative onset (methods mentioned in detail, i.e. including information about the method and/or steps taken beyond simply the name of the method: 12.0% pre vs. 19.8% post; OR 1.64, 95%CI 1.20-2.25); suicide in the headline:27.1% pre vs. 35.3% post; OR 1.30, 95%CI 1.07-1.58); suicide inevitable:0.2% pre vs. 8.4% post; OR 38.00, 95%CI 5.24-275.57;).

Results were also similar to the primary analysis for putatively protective content. The trend towards more articles with alternatives to suicide was now significant (12.7% pre vs. 18.0% post; OR 1.42, 95%CI 1.04-1.94).

Our secondary ARIMA model examined change across both the implementation and post-implementation periods (see Supplementary Figure 1). After controlling for confounders, mean monthly suicide deaths were unchanged (72.5±10.4 after vs. 63.9±9.4 before; RR 1.07, 95%CI 0.56-2.04; ω=5.44, SE=3.27, t=1.66, p=0.10). The Ljung Box showed no significant autocorrelations (Q=24.036; df=17; p=0.118) and the model accounted for 29% of the variation in monthly suicide deaths. Once again, population size and consumer price index were non-significant covariates; however, unemployment rate was significant (ω=-1.84, SE=0.58, t=-3.18, p=0.002).

Supplementary Table 1. Characteristics of articles focusing on suicide in major publications in Toronto media before and following release of the Mindset Guidelines.

| **Characteristics of media item** | **Total (%) *n* = 2447** | **Pre-initiative (%) *n* = 1250** | **Post-initiative (%) *n* = 1197** | **OR (95% CI)** |
| --- | --- | --- | --- | --- |
|  |  |  |  |  |
| **Item Location** |  |  |  |  |
| Print | 1308 (53.5) | 644 (51.5) | 664 (55.5) | 1.17 (1.0-1.37) |
|  |  |  |  |  |
| **Suicide Focus** |  |  |  |  |
| Ideation | 643 (26.3) | 325 (26.0) | 318 (26.6) | 1.03 (0.86-1.23) |
| Attempt | 399 (16.3) | 190 (15.2) | 209 (17.5) | 1.18 (0.95-1.46) |
| Death | 1749 (71.5) | 876 (70.1) | 873 (72.9) | 1.15 (0.96-1.37) |
|  |  |  |  |  |
| **Article Focus** |  |  |  |  |
| Specific person's death or suicidality | 1944 (79.4) | 1031 (82.5) | 913 (76.3) | 0.68 (0.56-0.83) |
| Suicide research | 470 (19.2) | 194 (15.5) | 276 (23.1) | 1.63 (1.33-2.00) |
| Suicide public policy | 453 (18.5) | 232 (18.6) | 221 (18.5) | 0.99 (0.81-1.21) |
| Assisted death | 571 (23.3) | 306 (24.5) | 265 (22.1) | 0.87 (0.72-1.05) |
| Individual murder-suicide | 134 (5.5) | 61 (4.9) | 73 (6.1) | 1.26 (0.89-1.79) |
| Mass murder-suicide | 51 (2.1) | 20 (1.6) | 31 (2.6) | 1.63 (0.92-2.88) |
| Suicide pact | 58 (2.4) | 31 (2.5) | 27 (2.3) | 0.90 (0.53-1.53) |
| Legal issues related to suicide | 1204 (49.2) | 627 (50.2) | 577 (48.2) | 0.92 (0.78-1.08) |
| Suicide in fiction | 134 (5.5) | 55 (4.4) | 79 (6.6) | 1.53 (1.07-2.18) |
|  |  |  |  |  |
| **Article type** |  |  |  |  |
| Opinion column | 445 (18.2) | 214 (17.1) | 231 (19.3) | 1.15 (0.94-1.42) |
|  |  |  |  |  |
| **Age Focus** |  |  |  |  |
| Youth | 709 (29.0) | 418 (33.4) | 291 (24.3) | 0.63 (0.53-0.76) |
| Adult | 815 (33.3) | 351 (28.1) | 464 (38.8) | 1.62 (1.36-1.92) |
| Older Adults | 221 (9.0) | 123 (9.8) | 98 (8.2) | 0.81 (0.61-1.07) |
|  |  |  |  |  |
| **Gender Focus** |  |  |  |  |
| Male | 1278 (52.2) | 623 (49.8) | 655 (54.7) | 1.21 (1.03-1.42) |
| Female | 1003 (41.0) | 569 (45.5) | 434 (36.6) | 0.68 (0.57-0.80) |

Supplementary Table 2: Putatively harmful and protective characteristics of articles focusing on suicide in Toronto media before and following release of the Mindset Guidelines.

| **Characteristics of media item** | **Total (%) *n* = 2447** | **Pre-initiative (%) *n* = 1250** | **Post-initiative (%) *n* = 1197** | **OR (95% CI)** |
| --- | --- | --- | --- | --- |
|  |  |  |  |  |
| **Putatively harmful** |  |  |  |  |
| Word "suicide" in the headline | 657 (26.8) | 338 (27.0) | 319 (26.6) | 0.98 (0.82-1.17) |
| Photo (deceased) | 464 (19.0) | 203 (16.2) | 261 (21.8) | 1.43 (1.17-1.76) |
| Photo (of someone looking sad) | 95 (3.9) | 33 (2.6) | 62 (5.2) | 2.01 (1.31-3.09) |
| Suicide method (in headline) | 81 (3.3) | 41 (3.3) | 40 (3.3) | 1.01 (0.65-1.58) |
| Suicide method (in text) | 1068 (43.6) | 557 (44.6) | 511 (42.7) | 0.92 (0.79-1.0) |
| Method described in detail | 253 (10.3) | 110 (8.8) | 143 (11.9) | 1.40 (1.08-1.82) |
| Favourable characteristic (deceased) | 266 (10.9) | 110 (8.8) | 156 (13.0) | 1.55 (1.20-2.01) |
| Statement that suicide is inevitable | 15 (0.6) | 3 (0.2) | 12 (1.0) | 4.20 (1.18-14.95) |
| Sensationalistic reporting | 73 (3.0) | 22 (1.8) | 51 (4.3) | 2.48 (1.49-4.12) |
| Glorified or romanticized suicide | 14 (0.6) | 8 (0.6) | 6 (0.5) | 0.78 (0.27-2.26) |
| Reasons for suicide (simplistic) | 234 (9.6) | 51 (4.1) | 183 (15.3) | 4.24 (3.07-5.85) |
| Identifies deceased as a celebrity | 230 (9.4) | 66 (5.3) | 164 (13.7) | 2.84 (2.11-3.83) |
| Interview with the bereaved | 450 (18.4) | 248 (19.8) | 202 (16.9) | 0.82 (0.66-1.00) |
|  |  |  |  |  |
| **Putatively protective** |  |  |  |  |
| Unfavourable characteristic (deceased) | 34 (1.4) | 16 (1.3) | 18 (1.5) | 1.17 (0.59-2.32) |
| Alternatives to suicide | 542 (22.1) | 198 (15.8) | 344 (28.7) | 2.14 (1.76-2.60) |
| Community resources | 55 (2.2) | 34 (2.7) | 21 (1.8) | 0.63 (0.36-1.10) |
| Positive outcome of a suicide-related crisis | 24 (1.0) | 14 (1.1) | 10 (0.8) | 0.74 (0.32-1.68) |
| Warning signs of suicidal behaviour | 16 (0.7) | 1 (0.1) | 15 (1.3) | 15.85 (2.09-120.18) |
| How to approach someone | 5 (0.2) | 1 (0.1) | 4 (0.3) | 4.18 (0.46-37.52) |
| Message of hope | 95 (3.9) | 42 (3.4) | 53 (4.4) | 1.33 (0.88-2.01) |

Supplementary Table 3. Characteristics of articles focusing on suicide in major publications in Toronto media before and following release of the Mindset Guidelines (excluding articles about the suicide of Robin Williams).

| **Characteristics of media item** | **Total (%) *n* = 2347** | **Pre-initiative (%) *n* = 1250** | **Post-initiative (%) *n* = 1097** | **OR (95% CI)** |
| --- | --- | --- | --- | --- |
|  |  |  |  |  |
| **Item Location** |  |  |  |  |
| Print | 1257 (53.6) | 644 (51.5) | 613 (55.9) | 1.19 (1.01-1.40) |
|  |  |  |  |  |
| **Suicide Focus** |  |  |  |  |
| Ideation | 634 (27.0) | 325 (26.0) | 309 (24.7) | 1.16 (0.93-1.33) |
| Attempt | 384 (16.4) | 190 (15.2) | 194 (15.5) | 1.19 (0.96-1.42) |
| Death | 1650 (70.3) | 876 (70.1) | 774 (61.9) | 1.02 (0.85-1.22) |
|  |  |  |  |  |
| **Article Focus** |  |  |  |  |
| Specific person's death or suicidality | 1846 (78.7) | 1031 (82.5) | 815 (65.2) | 0.61 (0.50-0.74) |
| Suicide research | 443 (18.9) | 194 (15.5) | 249 (19.9) | 1.59 (1.29-1.96) |
| Suicide public policy | 433 (18.4) | 232 (18.6) | 201 (16.1) | 0.98 (0.79-1.21) |
| Assisted death | 571 (24.3) | 306 (24.5) | 265 (21.2) | 0.98 (0.81-1.18) |
| Individual murder-suicide | 134 (5.7) | 61 (4.9) | 73 (5.8) | 1.39 (0.97-1.97) |
| Mass murder-suicide | 51 (2.2) | 20 (1.6) | 31 (2.5) | 1.78 (1.01-3.15) |
| Suicide pact | 58 (2.5) | 31 (2.5) | 27 (2.2) | 0.99 (0.58-1.67) |
| Legal issues related to suicide | 1204 (51.3) | 627 (50.2) | 577 (46.2) | 1.10 (0.93-1.29) |
| Suicide in fiction | 133 (5.7) | 55 (4.4) | 78 (6.2) | 1.66 (1.16-2.37) |
|  |  |  |  |  |
| **Article type** |  |  |  |  |
| Opinion column | 428 (18.2) | 214 (17.1) | 214 (17.1) | 1.17 (0.95-1.44) |
|  |  |  |  |  |
| **Age Focus** |  |  |  |  |
| Youth | 703 (30.0) | 418 (33.4) | 285 (22.8) | 0.69 (0.58-0.83) |
| Adult | 716 (30.5) | 351 (28.1) | 365 (29.2) | 1.27 (1.07-1.52) |
| Older Adults | 215 (9.2) | 123 (9.8) | 92 (7.4) | 0.83 (0.63-1.11) |
|  |  |  |  |  |
| **Gender Focus** |  |  |  |  |
| Male | 1179 (50.2) | 623 (49.8) | 556 (44.5) | 1.03 (0.87-1.21) |
| Female | 992 (42.3) | 569 (45.5) | 423 (33.8) | 0.75 (0.63-0.88) |

Supplementary Table 4: Putatively harmful and protective characteristics of articles focusing on suicide in Toronto media before and following release of the Mindset Guidelines (excluding articles about the suicide of Robin Williams).

| **Characteristics of media item** | **Total (%) *n* = 2347** | **Pre-initiative (%) *n* = 1250** | **Post-initiative (%) *n* = 1097** | **OR (95% CI)** |
| --- | --- | --- | --- | --- |
|  |  |  |  |  |
| **Putatively harmful** |  |  |  |  |
| Word "suicide" in the headline | 632 (26.9) | 338 (27.0) | 294 (26.8) | 0.98 (0.82-1.18) |
| Photo (deceased) | 405 (17.3) | 203 (16.2) | 202 (18.4) | 1.16 (0.94-1.44) |
| Photo (of someone looking sad) | 94 (4.0) | 33 (2.6) | 61 (5.6) | 2.17 (1.41-3.34) |
| Suicide method (in headline) | 77 (3.3) | 41 (3.3) | 36 (3.3) | 1.00 (0.63-1.57) |
| Suicide method (in text) | 1023 (43.6) | 557 (44.6) | 466 (42.5) | 0.91 (0.78-1.08) |
| Method described in detail | 239 (10.2) | 110 (8.8) | 129 (11.8) | 1.38 (1.05-1.80) |
| Favourable characteristic (deceased) | 200 (8.5) | 110 (8.8) | 90 (8.2) | 0.92 (0.69-1.23) |
| Statement that suicide is inevitable | 10 (0.4) | 3 (0.2) | 7 (0.6) | 2.66 (0.68-10.34) |
| Sensationalistic reporting | 69 (2.9) | 22 (1.8) | 47 (4.3) | 2.49 (1.49-4.17) |
| Glorified or romanticized suicide | 14 (0.6) | 8 (0.6) | 6 (0.5) | 0.85 (0.29-2.46) |
| Reasons for suicide (simplistic) | 232 (9.9) | 51 (4.1) | 181 (16.5) | 4.64 (3.36-6.41) |
| Identifies deceased as a celebrity | 133 (5.7) | 66 (5.3) | 67 (6.1) | 1.16 (0.82-1.65) |
| Interview with the bereaved | 427 (18.2) | 248 (19.8) | 179 (16.3) | 0.78 (0.63-0.97) |
|  |  |  |  |  |
| **Putatively protective** |  |  |  |  |
| Unfavourable characteristic (deceased) | 33 (1.4) | 16 (1.3) | 17 (1.5) | 1.21 (0.61-2.41) |
| Alternatives to suicide | 499 (21.3) | 198 (15.8) | 301 (27.4) | 2.00 (1.64-2.45) |
| Community resources | 49 (2.1) | 34 (2.7) | 15 (1.4) | 0.49 (0.26-0.91) |
| Positive outcome of a suicide-related crisis | 24 (1.0) | 14 (1.1) | 10 (0.9) | 0.81 (0.35-1.83) |
| Warning signs of suicidal behaviour | 13 (0.6) | 1 (0.1) | 12 (1.1) | 13.81 (1.79-106.40) |
| How to approach someone | 3 (0.1) | 1 (0.1) | 2 (0.2) | 2.28 (0.20-25.19) |
| Message of hope | 81 (3.5) | 42 (3.4) | 39 (3.6) | 1.06 (0.68-1.65) |

Supplementary Table 5. Characteristics of articles focusing on suicide in major publications in Toronto media before and following onset of a national media engagement initiative (November 2009-November 5, 2015 vs. November 6, 2015-October 2021).

| **Characteristics of media item** | **Total (%) *n* = 900** | **Pre-initiative (%) *n* = 450** | **Post-initiative (%) *n* = 450** | **OR (95% CI)** |
| --- | --- | --- | --- | --- |
|  |  |  |  |  |
| **Item Location** |  |  |  |  |
| Print | 504 (56.0) | 280 (62.2) | 224 (49.8) | 0.80 (0.71-0.90) |
|  |  |  |  |  |
| **Suicide Focus** |  |  |  |  |
| Ideation | 352 (39.1) | 105 (23.3) | 247 (54.9) | 2.35 (1.95-2.83) |
| Attempt | 326 (36.2) | 82 (18.2) | 244 (54.2) | 2.97 (2.40-3.68) |
| Death | 608 (67.6) | 325 (72.2) | 283 (62.9) | 0.87 (0.79-0.95) |
|  |  |  |  |  |
| **Article Focus** |  |  |  |  |
| Specific person's death or suicidality | 517 (57.4) | 352 (78.2) | 165 (36.7) | 0.46 (0.41-0.53) |
| Suicide research | 258 (28.7) | 96 (21.3) | 162 (36.0) | 1.68 (1.36-2.09) |
| Suicide public policy | 227 (25.2) | 70 (15.6) | 157 (34.9) | 2.24 (1.74-2.87) |
| Assisted death | 115 (12.8) | 91 (20.2) | 24 (5.3) | 0.26 (0.17-0.40) |
| Individual murder-suicide | 75 (8.3) | 40 (8.9) | 35 (7.8) | 0.87 (0.56-1.35) |
| Mass murder-suicide | 24 (2.7) | 9 (2.0) | 15 (3.3) | 1.66 (0.73-3.76) |
| Suicide pact | 17 (1.9) | 11 (2.4) | 6 (1.3) | 0.54 (0.20-1.46) |
| Legal issues related to suicide | 227 (25.2) | 171 (38.0) | 56 (12.4) | 0.32 (0.24-0.43) |
| Suicide in fiction | 31 (3.4) | 20 (4.4) | 11 (2.4) | 0.55 (0.26-1.13) |
|  |  |  |  |  |
| **Article type** |  |  |  |  |
| Opinion column | 95 (10.6) | 84 (18.7) | 11 (2.4) | 0.13 (0.07-0.24) |
|  |  |  |  |  |
| **Age Focus** |  |  |  |  |
| Youth | 249 (27.7) | 130 (28.9) | 119 (26.4) | 0.91 (0.74-1.13) |
| Adult | 209 (23.2) | 128 (28.4) | 81 (18.0) | 0.63 (0.49-0.80) |
| Older Adults | 54 (6.0) | 40 (8.9) | 14 (3.1) | 0.35 (0.19-0.63) |
|  |  |  |  |  |
| **Gender Focus** |  |  |  |  |
| Male | 451 (50.1) | 243 (54.0) | 208 (46.2) | 0.85 (0.75-0.97) |
| Female | 279 (31.0) | 157 (34.9) | 122 (27.1) | 0.77 (0.63-0.94) |

Supplementary Table 6: Putatively harmful and protective characteristics of articles focusing on suicide in Toronto media before and following onset of a national media engagement initiative (November 2009-November 5, 2015 vs. November 6, 2015-October 2021).

| **Characteristics of media item** | **Total (%) *n* = 900** | **Pre-initiative (%)*n* = 450** | **Post-initiative (%)*n* = 450** | **OR (95% CI)** |
| --- | --- | --- | --- | --- |
|  |  |  |  |  |
| **Putatively harmful** |  |  |  |  |
| Word "suicide" in the headline | 281 (31.2) | 122 (27.1) | 159 (35.3) | 1.30 (1.07-1.58) |
| Photo (deceased) | 85 (9.4) | 77 (17.1) | 8 (1.8) | 0.10 (0.05-0.21) |
| Photo (of someone looking sad) | 18 (2.0) | 14 (3.1) | 4 (0.9) | 0.28 (0.09-0.86) |
| Suicide method (in headline) | 43 (4.8) | 23 (5.1) | 20 (4.4) | 0.87 (0.48-1.56) |
| Suicide method (in text) | 327 (36.3) | 237 (52.7) | 90 (20.0) | 0.38 (0.31-0.46) |
| Method described in detail | 143 (15.9) | 54 (12.0) | 89 (19.8) | 1.64 (1.20-2.25) |
| Favourable characteristic (deceased) | 57 (6.3) | 44 (9.8) | 13 (2.9) | 0.29 (0.16-0.54) |
| Statement that suicide is inevitable | 39 (4.3) | 1 (0.2) | 38 (8.4) | 38.0 (5.2-275.6) |
| Sensationalistic reporting | 16 (1.8) | 10 (2.2) | 6 (1.3) | 0.60 (0.22-1.63) |
| Glorified or romanticized suicide | 9 (1.0) | 4 (0.9) | 5 (1.1) | 1.25 (0.33-4.62) |
| Reasons for suicide (simplistic) | 140 (15.6) | 39 (8.7) | 101 (22.4) | 2.59 (1.83-3.65) |
| Identifies deceased as a celebrity | 61 (6.8) | 41 (9.1) | 20 (4.4) | 0.48 (0.29-0.81) |
| Interview with the bereaved* | 141 (15.7) | 88 (19.6) | 53 (11.8) | 0.60 (0.44-0.82) |
|  |  |  |  |  |
| **Putatively protective** |  |  |  |  |
| Unfavourable characteristic (deceased) | 12 (1.3) | 6 (1.3) | 6 (1.3) | 1.00 (0.32-3.07) |
| Alternatives to suicide | 138 (15.3) | 57 (12.7) | 81 (18.0) | 1.42 (1.04-1.94) |
| Community resources | 156 (17.3) | 5 (1.1) | 151 (33.6) | 30.2 (12.5-72.9) |
| Positive outcome of a suicide-related crisis | 42 (4.7) | 4 (0.9) | 38 (8.4) | 9.50 (3.41-26.39) |
| Warning signs of suicidal behaviour | 122 (13.6) | 4 (0.9) | 118 (26.2) | 29.5 (11.0-79.2) |
| How to approach someone | 86 (9.6) | 1 (0.2) | 85 (18.9) | 85.0 (11.9-607.8) |
| Message of hope | 68 (7.6) | 19 (4.2) | 49 (10.9) | 2.57 (1.54-4.30) |

*Guidelines urge caution in reporting interviews with the bereaved but this may not necessarily be harmful.

Supplementary Table 7: Mention of specific methods in articles focusing on suicide in major publications in Toronto media before and following the national initiative.

| **Characteristics of media item** | **Total (%) *n* = 900** | **Pre-initiative (%) n=450** | **Post-initiative (%) n=450** | **OR (95% CI)** |
| --- | --- | --- | --- | --- |
|  |  |  |  |  |
| **Method of suicide** |  |  |  |  |
| Hanging | 78 (8.7) | 62 (6.9) | 16 (1.8) | 0.25 (0.15-0.44) |
| Overdose | 48 (5.3) | 35 (3.9) | 13 (1.4) | 0.37 (0.19-0.69) |
| Jumping (building) | 16 (1.8) | 12 (1.3) | 4 (0.4) | 0.33 (0.10-1.02) |
| Jumping (bridge) | 26 (2.9) | 21 (2.3) | 5 (0.6) | 0.23 (0.09-0.62) |
| Railway | 14 (1.6) | 13 (1.4) | 1 (0.1) | 0.07 (0.01-0.58) |
| Car exhaust | 1 (0.1) | 1 (0.1) | 0 (0.0) | 1.00 (0.99-1.00) |
| Other asphyxia | 27 (3.0) | 27 (3.0) | 0 (0.0) | 1.06 (1.03-1.08) |
| Firearm | 90 (10.0) | 60 (6.7) | 30 (3.3) | 0.50 (0.32-0.76) |
| Suicide by cop | 12 (1.3) | 3 (0.3) | 9 (1.0) | 3.00 (0.81-11.00) |
| Cutting or stabbing | 17 (1.9) | 12 (1.3) | 5 (0.6) | 0.41 (0.14-1.17) |
| Drowning | 8 (0.9) | 7 (0.8) | 1 (0.1) | 0.14 (0.01-1.15) |
| Lethal injection | 17 (1.9) | 17 (1.9) | 0 (0.0) | 1.03 (1.02-1.05) |
| Burning or electrocution | 8 (0.9) | 1 (0.1) | 7 (0.8) | 7.00 (0.86-56.66) |
| Other | 31 (3.4) | 15 (1.7) | 16 (1.8) | 1.06 (0.53-2.13) |

Supplementary Figure 1. Monthly suicides in the GTA, November 2009-April 2021. *Note*. The solid line represents the month in which the multi-pronged initiative to engage media was initiated.
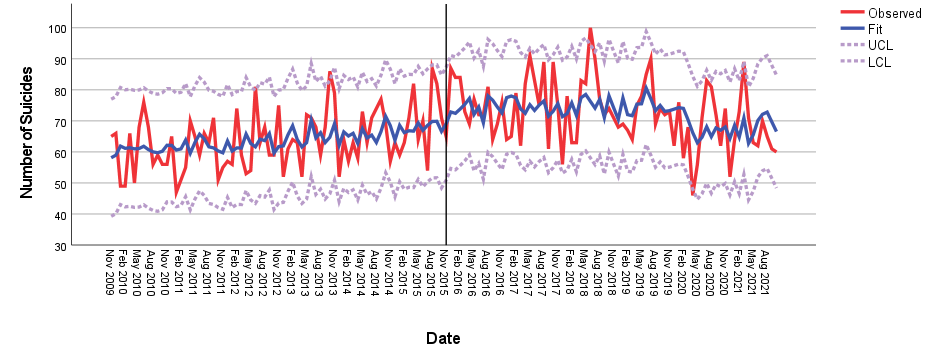


*UCL: Upper control limit; LCL: Lower control limit
